# Economic Evaluation of the InTENSE Program of Therapy Alongside Botulinum Neurotoxin a for the Rehabilitation of Chronic Upper Limb Spasticity

**DOI:** 10.3390/toxins17070341

**Published:** 2025-07-04

**Authors:** Rachel Milte, Jia Song, Sean Docking, Julie Ratcliffe, Ian D. Cameron, Maria Crotty, Louise Ada, Coralie English, Natasha A. Lannin

**Affiliations:** 1 Caring Futures Institute, Flinders University, Adelaide, SA 5001, Australia; jia.song@flinders.edu.au (J.S.); julie.ratcliffe@flinders.edu.au (J.R.); 2 School of Public Health and Preventive Medicine, Monash University, Melbourne, VIC 3004, Australia; sean.docking@monash.edu; 3 Sydney School of Health Sciences, University of Sydney, Sydney, NSW 2006, Australia; ian.cameron@sydney.edu.au (I.D.C.); louise.ada@sydney.edu.au (L.A.); 4 College of Medicine and Public Health, Flinders University, Adelaide, SA 5001, Australia; maria.crotty@flinders.edu.au; 5 School of Health Sciences, University of Newcastle, Callaghan, NSW 2308, Australia; coralie.english@newcastle.edu.au; 6 Department of Neuroscience, Monash University, Alfred Health, Melbourne, VIC 304, Australia; natasha.lannin@monash.edu

**Keywords:** stroke, rehabilitation, botulinum neurotoxin A, economic evaluation, cost–utility analysis, cost-effectiveness analysis, costs

## Abstract

Spasticity is a persistent and debilitating consequence of stroke and effective rehabilitation is a healthcare priority. Botulinum neurotoxin A (BoNT-A) with supportive therapy has increasingly been embedded within clinical practice for treatment of post-stroke spasticity. But the evidence for this approach has hitherto been limited to the findings of a limited number of small trials. The InTENSE trial was undertaken specifically to provide high-quality clinical trial evidence focusing on the effect of BoNT-A and adjunctive therapy on upper limb spasticity. While the clinical trial did not detect a significant impact upon clinical outcomes, there remains a need to evaluate any impact on the broader use of healthcare resources and overall cost-effectiveness. A detailed cost–utility analysis of the InTENSE trial was undertaken. The costs over the 12-month follow-up period were compared with quality-adjusted life years (QALY) gained using utilities generated from the EQ-5D three level (EQ-5D-3L) instrument. There were no significant differences in QALY gained between the intervention and control groups identified, or in the majority of health and community care costs. The Incremental Cost-Effectiveness Ratio per QALY gained was estimated at AU $63,947.11 (Australian dollars), which is well above accepted thresholds for cost-effectiveness in Australia. The study was unable to identify evidence for the cost-effectiveness of treatment approaches combining BoNT-A with adjunctive therapy.

## 1. Introduction

Stroke is among the top 10 leading causes of disability-adjusted life years lost in countries across the world [1]. One significant impact of a stroke can be spasticity, which has large implications for independence in activities of daily living for the person, and on caregiver burden for informal carers [2]. People living with spasticity experience significantly impaired quality of life compared to people post-stroke but not experiencing spasticity, and it commonly becomes a chronic condition requiring long-term management. Spasticity refers to a state of increased muscle tone with exaggerated reflexes and affects over 40% people following stroke [3]. It may impact only one part of a limb (known as focal) or can impact a range of areas of a limb or more than one limb itself. Treatment must, therefore, be individualised to target the combination of symptoms and their impact on an individual. While the effects of other deficits post-stroke, such as muscle weakness and loss of dexterity, can be significant, it is often muscle spasticity that has the most severe impact on an individual’s daily life, their independence, and engagement with the community. Additionally, spasticity can also be present with other complex post-stroke impairments, such as neglect, fatigue, and sensory or cognitive impairments. Therefore, long-term care can be complex and require input from multiple specialist health and allied health disciplines to achieve desired outcomes.

Effective treatments to maximise recovery of function following stroke, including for those experiencing spasticity, are crucial in practice but have been challenging to identify and translate into practice [4,5,6]. In the last decade, botulinum neurotoxin A (BoNT-A) with adjunctive therapy (i.e., treatment from a multidisciplinary therapy team including physiotherapy and occupational therapy primarily, with support from other health professionals such as orthotists and rehabilitation nurses as appropriate) has been embedded within national and international guidelines as treatment for post-stroke spasticity across the world [7,8]. Potentially, if the adjunctive therapy is successful in supporting the practice of beneficial movement patterns, strengthening abnormal muscle weakness, or reducing muscle overactivity in the short window post-injection during which the BoNT-A reduces muscle overactivity, the benefit from the injection could be maximised, for a relatively small additional cost. However, the evidence supporting this approach is limited, and recommendations have been based on expert opinion and extrapolated from a small number of trials rather than more high-quality clinical trials [7,9]. The InTENSE trial was conducted to evaluate the impact of BoNT-A treatment with specifically developed evidence-based adjunctive therapy compared with BoNT-A alone. This study focused on treatment of upper limb spasticity to ensure consistency in the outcome measures used across the sample. The clinical trial analysis found that the intervention did not have an effect on the primary or secondary clinical outcomes (in terms of the Goal Attainment Scale and the upper limb activity measured through the Box and Block Test) at 3 months [10,11], and a small gain in strength for the intervention group at 3 months was not maintained at the long-term 12-month follow-up [10]. However there remains a need to understand any impact on the use of healthcare resources and costs associated with the intervention, to inform a broader understanding of the cost-effectiveness of the InTENSE intervention.

Previous studies of the cost-effectiveness of botulinum neurotoxin A with or without adjunctive therapy have been mixed. Some previous cost-effectiveness studies indicated that use of Botox compared to no treatment is cost-effective, for example, Rychilk and Turcu [12,13]. However, it is relatively uncommon in most countries now that no treatment is offered to people with spasticity following stroke. Other studies have compared the use of botulinum toxin plus adjunctive therapy (such as movement training or physiotherapy) compared with adjunctive therapy alone. Studies have shown that the use of botulinum toxin and adjunctive therapy in the UK and Spain, compared with adjunctive therapy alone, was cost-effective [14,15,16]. In these studies, estimations were calculated using Markov or other models for post-stroke spasticity in general (i.e., upper or lower limbs or other sites). By comparison the BoTULS trial included a cost-effectiveness analysis conducted alongside their randomised clinical trial of botulinum toxin with an adjunctive therapy program compared with the toxin alone among those experiencing upper limb spasticity only [17]. The identified high costs in their treatment group and only small improvements in quality-adjusted life years (QALY) gained over a 3-month follow-up period from the health- and social care service perspective, and concluded the treatment was very unlikely to be cost-effective within the UK [17]. In an Australian-based study, Moore et al. [16] used a Markov model to estimate the cost-effectiveness of Abobulinumtoxin A and best supportive care compared with best supportive care alone for lower limb spasticity following stroke or traumatic brain injury. They identified an incremental cost-effectiveness ratio (ICER) of AU $35,721 per QALY gained, which was higher than previously estimated willingness-to-pay threshold of AU $28,000 per QALY gained in Australia [18]. A number of Markov models have been used to estimate the cost-effectiveness of newer forms of botulinum toxin A, such as incobotulinumtoxin A (Xeomin^®^, Merz Pharmaceuticals, Frankfurt am Main, Germany) or Abobotulinumtoxin A, which are similarly effective but currently cheaper and have less wastage than the alternative (Onabotulinumtoxin A or Botox^®^, Allergan Aesthetics, Dublin, Ireland), finding these to produce improved quality of life and/or reduced costs for the UK, Greek, or US health systems for treatment of spasticity and other conditions [15,16,17,19,20,21]. For Australia, Makino et al. [22] used a Markov model to estimate the cost-effectiveness of extending beyond the current limit of four treatment cycles of botulinum neurotoxin to unlimited treatment for those with post-stroke spasticity of an upper limb exhibiting continued adequate response to previous cycles [22]. They defined an adequate response as a greater than 1-point improvement in the Modified Ashworth Scale score (a common measure of spasticity). They estimated an ICER of AU $59,911 per QALY gained for the extended treatment program compared to the current treatment over a five-year period. Their model assumed a relatively high (although based on a previous clinical trial [23]) initial rate of adequate response to the Botox of 68.5%, followed by a 70% and 87.2% response rate in subsequent treatments for the previous responders.

There has been no evaluation of the cost-effectiveness of botulinum toxin A and adjunctive therapy compared with botulinum toxin A alone, particularly among those living with spasticity of an upper limb. Secondly, some Markov models produced used assumed effects on quality-of-life-improving utilities by 0.1 to 0.2 points and response rates of 60 to 70% among populations [12,14,16,20]. How realistic these assumptions are in the context of upper limb spasticity post-stroke in practice is unclear, where the extent of the disability experienced and the rate of recovery have hitherto been poor.

This study reports the findings of the cost–utility analysis and cost-effectiveness analysis of BoNT-A combined with an evidence-based adjunctive therapy treatment compared with Botox alone for people living with upper limb spasticity, alongside the InTENSE blinded-randomised controlled trial.

## 2. Materials and Methods

The clinical protocol for the trial, and the protocol for the economic evaluation have both previously been published in detail [24,25]. In brief, the economic evaluation was conducted alongside a Phase III clinical trial that aimed to determine the effectiveness of undertaking evidence-based movement training following a botulinum toxin A (BoNT-A) injection in adults with neurological spasticity. All participants received a program of injections with BoNT-A according to standard Australian practice recommendations, which, among other approaches, recommends guided injections to confirm the injection into the intended muscles [8]. The BoNT-A was supplied through the Pharmaceutical Benefits Scheme. The InTENSE program was administered to the intervention group by trained physical or occupational therapists immediately following their initial injection [25]. It included serial casting of the wrist, placing it in maximum extension for two weeks, followed by 10 weeks of movement training to reduce weakness and improve active movement (via a combination of electrical stimulation and progressive resistance exercises) and an intensive program of home exercises [26]. Participants received the intervention via a mix of in-clinic sessions, home visits, and phone calls for support.

### 2.1. Measurement, Valuation, and Analysis of Costs

A summary of the resources measured as part of the study, the cost values applied, and their sources are outlined in Table 1.

All costs were updated to a standard reference year for analysis. Discounting was not undertaken as the length of the study was 1 year. Total costs of care for the participants in the intervention and control groups over the 12-month follow-up period were calculated. These were aggregated into intervention costs, total healthcare costs (including costs of hospitalisation, emergency department presentations, doctors’ attendances, and visits to allied health professionals). Two participants passed away within the 12-month follow-up period; both were allocated to the intervention group. However, no accounting for censoring was undertaken as the proportion of total observations missing due to censoring was less than 1% [34].

### 2.2. Measurement and Valuation of Benefit

The primary economic evaluation framework applied was the cost–utility analysis (CUA). This approach was chosen for a number of reasons. Firstly, the trial provided two clear alternatives for comparison (a fundamental requirement for undertaking a CUA) [35]. Additionally, the potential benefits of the intervention were expected to be across multiple clinical domains (e.g., spasticity, pain, function, physical and mental wellbeing) rather than a single clearly defined clinical domain. Therefore, measuring the benefits of the intervention using the concept of quality-adjusted life years (QALY), which incorporates benefits across a number of areas of a person’s life simultaneously, was considered most appropriate for this study, leading to the selection of the CUA framework. The benefit of the intervention was primarily measured via quality-adjusted life years (QALY) gained by participants over the 12-month follow-up period. Health-related quality of life (HRQoL) was measured using the EQ-5D-3L instrument [36]. The EQ-5D-3L measures HRQoL across 5 dimensions using three levels of possible response for each dimension. Participants were asked to rate their HRQoL during the last week using the EQ-5D-3L, at baseline, 3-month follow-up, and 12-month follow-up. These responses were then used to calculate the individual utility scores for participants using the preference-based scoring algorithm developed for Australia [37]. The utility scores created are on a scale where 0 represents a health state equivalent to death, 1 represents full health, and negative values are possible indicating a health state considered worse than death. These individual utility scores were then combined with the time that participants spent in each of these health states and used to calculate the overall QALY gain for participants using the area-under-the-curve method [35].

A secondary analysis was undertaken using the Goal Attainment Scale (GAS) scores over the 12-month follow-up period as the measure of benefit in a cost-effectiveness analysis (CEA). The GAS is a validated measure of achievement of rehabilitation specific goals identified by the participants themselves, usually related to activity and participation in meaningful tasks, and was chosen as it is a recommended outcome measure in Australian Clinical Guidelines [8]. Participants then score themselves for current and expected levels of performance (ranging from −2 to +2), with t-scores then calculated as described by Kiresuk et al. [38].

### 2.3. Cost–Utility Analysis

The economic evaluation was undertaken on an intention to treat basis. The difference in healthcare costs between the intervention and control groups was divided by the difference in QALY gain to give an incremental cost-effectiveness ratio (ICER) of cost per QALY gained (i.e., ICER = Ca − Cb/Ea − Eb, where Ca is the cost of the intervention, Cb is the cost of the control, Ea is the effectiveness of the intervention, and Eb is the effectiveness of the control). We also planned to undertake a subgroup analysis using those participants with some function of the upper extremity at baseline (i.e., those who could move 1 or more blocks on the Box and Block test at baseline). However, given only 31 participants met this criterion, we did not perform the subgroup analysis.

### 2.4. Statistical Analysis

A statistical analysis was undertaken using Stata version 17.0. Although cost and quality-of-life data were skewed (as is common and well described with this type of data), an approach reporting mean values was utilised, as has been recommended previously. Missing data on costs and selected outcomes were addressed using multiple imputation by chained equations (MICE) in Stata (version 17.0), implemented in wide format. MICE specifies an imputation model for each variable, iteratively predicting missing values using the available data until convergence is achieved [39]. The imputation model targeted missing values in monthly cost data (diary costs from month 5 to month 11) and two outcome variables (EQ-5D-3L scores at 3 months and 12 months). The number of complete cases available for analysis is presented by group allocation in Supplementary Information Table S1. In summary, 132 participants had complete data for EQ-5D-5L at the 12-month time point, 134 had complete Medicare Benefits Schedule (doctor’s appointments, laboratory tests, radiology, and allied health appointments) and Pharmaceutical Benefit Scheme (prescription pharmaceuticals) data (which comprises the majority of community-based healthcare in Australia [40]). However, completion of the monthly diary was much lower, declining from 99 to 57 of participants over time, necessitating the focus on this in the imputation model. We used predictive mean matching (PMM) with 12 nearest neighbours to account for the non-normal distribution of cost data. Thirty imputations were created using 300 iterations with a 10-iteration burn-in. The following demographics and clinical baseline characteristics variables were included as predictors: age, gender, centre of treatment, ambulation status, mental health status, baseline Tardieu scale at wrist, baseline Box and Block test score, days since stroke, and baseline EQ-5D score. The stability and convergence of the imputation model were assessed using trace plots, comparisons between observed and imputed distributions, and missing data diagnostics. All analyses were conducted using mi estimate, and final cost-effectiveness results were pooled across the 30 imputed datasets using Rubin’s rules [41] to account for both between-imputation and within-imputation variance components associated with the estimates.

Following imputation, mean incremental costs and mean incremental quality-adjusted life years (QALY) were calculated using Seemingly Unrelated Regression (SUR) models to account for the correlation between costs and outcomes [42]. Non-parametric bootstrap methods with 10,000 replications were also employed to generate the joint distribution of costs and outcomes, which were used to populate the cost-effectiveness plane and cost-effectiveness acceptability curve.

## 3. Results

As has been previously reported, 140 people with stroke were recruited to the study (69 randomised to the intervention and 71 to the control group) [11]. There were no significant differences between the intervention and control groups at baseline in any demographic or clinical characteristic tested for (see Table 2).

Table 3 provides the mean utility scores for the two groups including both the raw and the imputed values. At baseline, the raw utility scores derived from the responses to the EQ-5D-3L were similar (0.04 difference between the two in favour of the intervention group). At 12 months, while both groups improved in their utility scores, the difference remained consistent between the two groups (0.03) and not statistically significant. Table 4 gives the mean use of Australian Medical Benefits Schedule (MBS) and Australian Pharmaceutical Benefits Scheme (PBS) resources for the participants in the two groups over the 12-month trial period. There were no significant differences in the use of these resources over the trial period and the control group also used substantial resources. Table 5 gives the costs for the health- and social care services used by participants over the 12-month trial period. As expected, the intervention group incurred significantly higher costs as part of the intervention. But apart from this, there were no significant differences in costs of health service use between the two groups, excluding for use of podiatry, where there was an increased cost among the intervention group (difference AU $96.80, *p* = 0.047).

Table 6 gives the results of the base case costs and QALY gain over the 12-month trial period for both groups, and the ICER for the final imputed values. The intervention group incurred significantly higher total costs over the 12-month trial period compared to the control (difference AU $5111.04, *p* = 0.02). However, there was no significant difference between the two groups in QALY gain. This resulted in a ICER of AU $63,974 per QALY gained for the InTENSE program, a value well above the estimated WTP for a QALY in Australia of AU $28,000 [18]. A sensitivity analysis was undertaken using only cases with complete data (n = 53) with a similar finding (ICER of AU $65,792.80).

Table 6 also gives the results of the secondary analysis using the GAS as the measure of benefit across the 12-month follow-up period. Similar to the findings with QALY as a measure of benefit, there were not significant differences in GAS scores over the 12-month time period using either the imputed values or when considering only complete cases (sensitivity analysis). The estimated ICER for this analysis was AU $1,667.51 per point improvement in GAS between the two groups for the imputed values, with a similar finding (AU $1,563.76) when only complete cases were considered.

**Figure 1:**
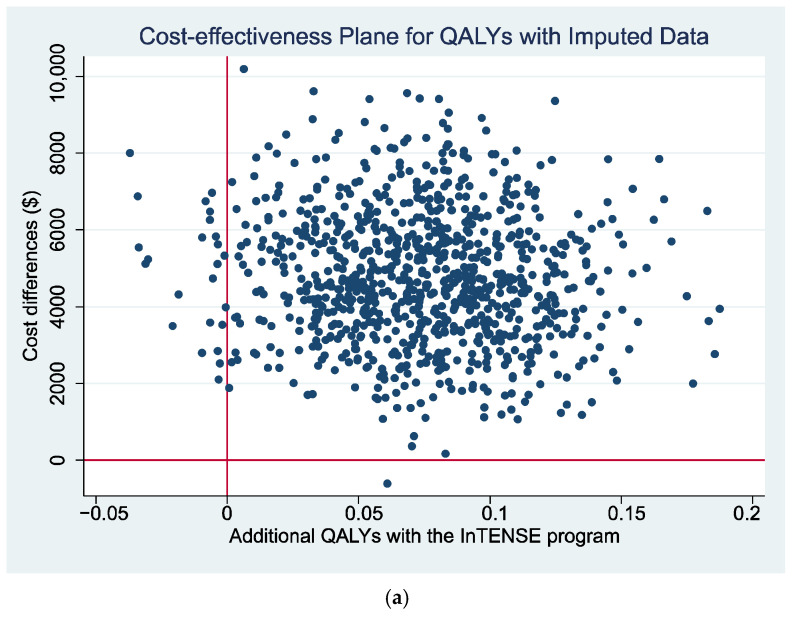

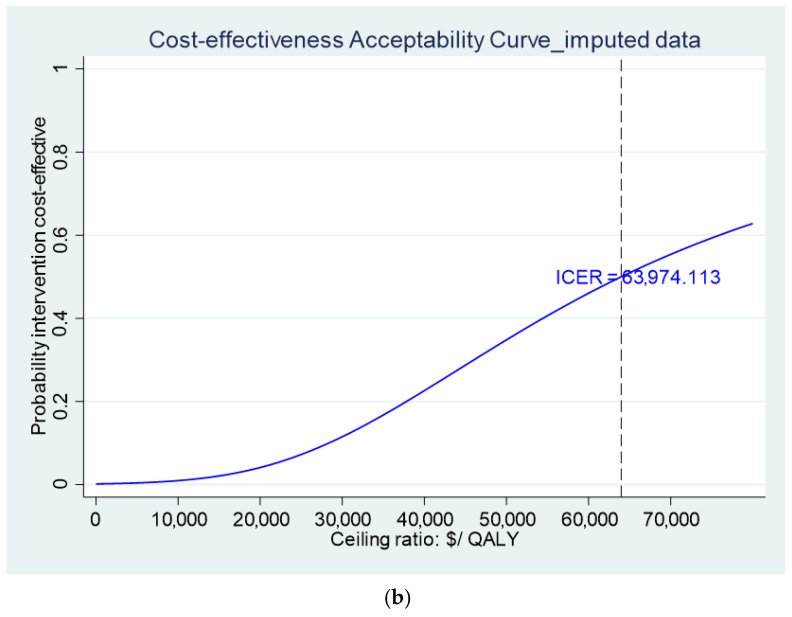
(**a**) Cost-effectiveness plane and (**b**) CEAC using QALYs gain over 12 months as measure of benefit.

**Figure 2:**
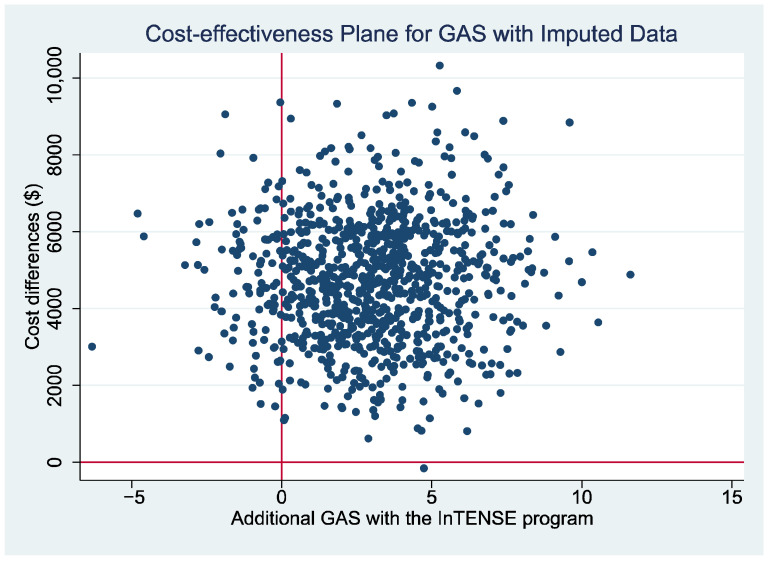
Cost-effectiveness plane using difference in GAS score between the intervention and usual care groups at 12 months as measure of benefit.

The cost-effectiveness plane presenting the non-parametric bootstrapped estimates of the ICER is presented for the estimates with the QALY gain as the measure of benefit (Figure 1a) and with the GAS as the measure of benefit (Figure 2) as a graphical representation of the uncertainty around the estimates of the ICER. It can be seen that, for both figures, there is a relatively large scatter of points in the top north-east quadrant of the plane (which indicates the intervention is more effective but also more costly than the control) across into the north-west quadrant of the plane (indicating that the intervention is less effective than the control and also more costly). The relatively large spread of data points indicates there is currently a large amount of uncertainty around the point estimates of the ICER (supported by wide 95% CI reported in Table 6). Additionally, a cost-effectiveness acceptability curve (CEAC) is presented representing the probability that the intervention would be considered cost-effective compared to the control for a range of potential values of cost-effectiveness thresholds for the primary analysis (i.e., ICER cost/QALY gained over 12 months). This figure indicates a relatively low likelihood of cost-effectiveness across the range of presented thresholds, reaching only just over 60% probability at a willingness-to-pay threshold of AU $70,000 per QALY gained, well beyond accepted thresholds in Australia. The figures displaying the cost-effectiveness plane and CEAC for the complete case data are presented in Supplementary Materials (Figures S1 and S2).

## 4. Discussion

This study reports the findings of a detailed economic evaluation alongside the InTENSE clinical trial [9,10] applying a cost–utility and cost-effectiveness analysis framework. Significantly higher costs related to the provision of the intervention were identified, but no significant differences were observed in other health- and social care costs for the sample of people living with moderate or severe upper limb spasticity post-stroke. The limited improvement in HRQOL over the 12-month follow-up period across both groups, and the lack of significant difference between the groups, resulted in a relatively high estimated ICER (over AU $63,000), which is above the current estimated WTP fora QALY in Australia (AU $28,000) [18].

Previous studies evaluating the cost-effectiveness of BoNT-A are relatively few, and none currently provide a direct comparison to the intervention and control in this study. Within the Australian setting, Makino et al. [22] undertook a cost-effectiveness analysis of extending access to BoNT-A funded by the Pharmaceutical Benefits Scheme in Australia among people with upper limb spasticity beyond the currently approved four treatment cycles for those who have an adequate response to previous treatment cycles. They concluded that the extension in access may be cost-effective if provided to patients most likely to continue to see treatment gains. They estimated an ICER per QALY gained for the extended treatment among those who had demonstrated a successful response to previous treatment of AU $59,911. It should be noted that, similar to the current study, the ICER for this study was well above current estimates of willingness to pay for aQALY in Australia. This study also differed from the current study, which was based on data collected directly alongside a clinical trial. Instead, it was estimated using a Markov-state transition model developed using estimated treatment effects from previously published studies and, in this case, predominantly from a single randomised controlled trial and extension study conducted in Germany [23]. Additionally, this model was based on around 70% of patients improving following treatment with BoNT-A. However, we did not see this level of improvement in either group in the InTENSE trial [10].

In comparison, while the current study identified a similar ICER per QALY gained, cost-effectiveness cannot be concluded given it is significantly above current published estimates of willingness to pay (WTP) for a QALY for Australia. The current study also did not show any impact on health- or community care costs between the two groups, which if it did occur, could have been considered a benefit to the Australian taxpayer, who bears the vast majority of the costs of providing this intervention. This study is, therefore, unable to recommend this intervention as value for money for the Australian health system currently for people in the chronic phase after stroke with limited or no function in the affected upper extremity. The use of BoNT-A only (the control group) is also costly and demonstrated no clinical efficacy [43].

This economic evaluation does have some limitations, which should be considered alongside the findings. While the study was able to include a wide variety of health and community care costs incurred for participants, it did not collect information on informal carer costs (for example, informal carer time providing assistance with daily tasks, loss of productivity from informal carers leaving the workforce, etc.). While informal carer costs have typically been given haphazard consideration by health economists in the past, in recent years, the significant economic impact of informal caring has been recognised. There is significant support provided by carers that the current social care system simply would be incapable of providing. In addition, there is increasing acknowledgement of inequity in the impact of informal caregiving. A large proportion of informal care is provided by women, who, if they left the workforce to provide this care, potentially experience a reduction in income, not only at the time of providing care, but via follow-on effects through reduced contribution to retirement funding (which in Australia at this time is substantially supported through superannuation contributions accrued over time in the workforce). Additionally, there is also the potential for an intervention to have an impact on the quality of life and wellbeing of informal carers. While traditionally, CUA has focused heavily on the impact on the individual participants, more recently, a model for incorporating ‘spillovers’ from the individual participant to their family networks has been proposed [44]. Future economic evaluations should consider informal caregiver costs and impact on quality of life and wellbeing to provide greater understanding of the potential impact of interventions on informal caregivers, to give a true societal perspective of cost-effectiveness. Additionally, we found relatively low completion rates of monthly participant cost diaries in our sample and this completion declined over time. Other studies have also experienced challenges in collecting self-report healthcare utilisation data, particularly over long periods of time [45,46]. However, general engagement with the trial follow-up measures in the current trial was excellent, with over 94% of participants completing follow-up assessments at 3 and 12 months, and very low levels of missing data for administrative linked data. This provides data on the majority of community healthcare, which in Australia is government-funded through the Medicare Benefits Schedule and Pharmaceutical Benefits Scheme. Future studies should continue to prioritize the use of administrative linked datasets for the collection of healthcare costs in Australia [40].

## 5. Conclusions

In conclusion, this study was unable to find evidence for the cost-effectiveness of BoNT-A combined with adjunctive therapy, compared with BoNT-A only, in a population of people living with upper limb spasticity and limited upper limb function post-stroke.

## Figures and Tables

**Table 1:** Unit costs and their sources.

Cost	Value	Source of Data	Source of Unit Cost
Intervention therapist time	AU $67.95 for 30 min consultation	Intervention records	Australian Department of Veterans’ affairs [27]
Intervention therapist travel	AU $0.72 per km	Intervention records	Australian Taxation Office
Intervention consumables (e.g., casts)	AU $95 GRASP kit AU $255.45 electrical stimulation machine AU $14.99 per hard/soft cast roll AU $23.67 per padding roll	Intervention records	Hospital finance data
Doctor visits Laboratory tests Radiology	Various	Administrative linked data	Medicare Benefits Schedule [28]
Medications	Various	Administrative linked data	Pharmaceutical Benefits Scheme [29]
Inpatient admissions	AU $5324	Participant monthly diary	National Hospital Cost Data Collection Round 23 [30]
Emergency department presentations	AU $775	Participant monthly diary	National Hospital Cost Data Collection Round 23
Allied health outpatient visits	Various	Participant monthly diary	Department of Veterans’ Affairs
Home aged care services	AU $29–170 per day	Participant monthly diary	My Aged Care, Commonwealth Home Support Program [31,32]
Residential aged care services	AU $229.43 per day	Participant monthly diary	Department of Health- and Aged Care [33]

**Table 2:** Baseline characteristics.

Variable	Intervention	Control	Test of Difference
Age (in years)	61.81	60.18	ns
Gender (female)	0.32	0.30	ns
Time since stroke (days)	59.46	66.76	ns
Side of hemiplegia (Right)	0.35	0.36	ns
Can walk on own	0.42	0.46	ns
Short Portable Mental Status Questionnaire (0–10)	8.94	9.11	ns
Level of education	0.19	0.30	ns
Living situation	0.19	0.15	ns
Sensation (0–2)	1.01	0.92	ns
Neglect (0–3)	0.22	0.28	ns
Box and Block Test (number of valid boxes)	1.67	2.15	ns
Pain scale (0–111 mm)	1.91	1.86	ns

Note: ns—not statistically significant.

**Table 3:** Mean outcomes per participant.

Variable	Intervention (SD)	Control (SD)	Difference (Bootstrapped 95% CI), *p*-Value
*EQ-5D-3L Scores* (*Raw* (*unimputed*) *scores*)			
EQ-5D-3L Scores at baseline	0.54 (0.03)	0.50 (0.03)	0.04, [−0.04, 0.13], *p =* 0.310
EQ-5D-3L Scores at 3 months	0.60 (0.03)	0.54 (0.03)	0.06, [−0.02, 0.14] *p =* 0.137
EQ-5D-3L Scores at 12 months	0.60 (0.03)	0.57 (0.03)	0.03, [−0.06, 0.11], *p =* 0.516
*EQ-5D-3L Scores* (*imputed values*)			
EQ-5D-3L Scores at 3 months ^a^	Not imputed		
EQ-5D-3L Scores at 12 months ^b^	0.60 (0.25)	0.57 (0.24)	0.03 [−0.05, 0.12] *p =* 0.458
*Goal Attainment Scale* (*GAS*) *Scores* (*unimputed*)			
GAS scores at 3 months	43.19 (12.37)	40.74 (12.15)	2.44 [−1.76, 6.64] *p =* 0.254
GAS scores at 12 months	41.22 (14.33)	41.00 (13.47)	0.22 [−4.69, 5.12] *p =* 0.930

Notes: ^a^: missing rate = 1%; ^b^: missing rate = 5.7%.

**Table 4:** Mean MBS and PBS resource use per participant over 12 months (raw non-imputed values).

Resources	Intervention (SD)	Control (SD)	Difference (Bootstrapped 95% CI), *p*-Value
GP visits	9.91 (1.25)	12.03 (1.64)	−2.12 [−6.28, 2.04] *p* = 0.318
Imaging procedures	1.58 (0.26)	2.43 (0.47)	−0.85 [−1.89, 0.19] *p* = 0.108
Diagnostic procedures	0.58 (0.15)	0.74 (0.16)	−0.16 [−0.57, 0.25] *p* = 0.450
Miscellaneous procedures	2.39 (0.34)	3.47 (0.48)	−1.08 [−2.19, 0.04] *p* = 0.058
Pathology procedures	13.42 (2.14)	15.84 (3.97)	−2.41 [−11.56, 6.73] *p* = 0.605
Professional Attendances	8.55 (1.59)	9.66 (1.33)	−1.12 [−4.99, 2.76] *p* = 0.572
Therapeutic procedures	1.06 (0.24)	1.63 (0.37)	−0.57 [−1.45, 0.31] *p* = 0.202
BoNT-A injection procedures	0.30 (0.09)	0.28 (0.09)	0.02 [−0.22, 0.27] *p* = 0.850
Drug prescriptions	57.80 (4.73)	64.01 (5.22)	−6.21 [−19.67, 7.24] *p* = 0.366

GP—general practitioner.

**Table 5:** Mean costs (Australian dollars) per participant over 12 months (complete cases only, no imputations).

Costs (AU $)	Intervention (SD)	Control (SD)	Difference (Bootstrapped 95% CI)
*Intervention costs*	3249.35 (1032.01)	0 (0)	3249.35 [2997.68, 3501.03] *p =* 0.000
*Hospital*			
In-hospital	1746.80 (3007.70)	1080.15 (2341.90)	666.65 [−240.58, 1573.89] *p* = 0.150
Emergency department	147.65 (364.58)	112.35 (305.35)	35.31 [−80.24, 150.86] *p* = 0.549
*Community care*			
OT and PT	654.32 (1886.98)	264.62 (1122.17)	389.69 [−134.36, 913.75] *p* = 0.145
Podiatry	114.18 (395.04)	17.38 (93.57)	96.80 [1.12, 192.48] *p* = 0.047
Home nursing	1724.67 (10532.42)	605.26 (3193.52)	1119.42 [−1362.94, 3061.77] *p* = 0.377
Domestic assistance	910.29 (2723.25)	434.12 (1434.98)	479.16 [−291.49, 1243.82] *p* = 0.224
Residential aged care	5281.11 (14427.76)	2992.57 (10255.17)	2288.54 [−1986.17, 6563.25] *p* = 0.294
*MBS and PBS*	2227.28 (1696.59)	2962.91 (3401.55)	−735.64 [−1620.42, 149.14] *p* = 0.103
General Practitioner	521.99 (456.75)	662.57 (860.83)	−140.59 [−364.48, 83.31] *p =* 0.218
Imaging	244.75 (388.34)	326.94 (494.74)	−82.19 [−236.56, 72.18] *p =* 0.297
Diagnostic procedures	40.76 (89.66)	56.42 (111.51)	−15.67 [−49.07, 17.74] *p =* 0.358
Miscellaneous procedures	150.02 (247.08)	202.86 (259.67)	−52.84 [−136.46, 30.77] *p =* 0.215
Pathology	221.49 (296.88)	272.82 (508.53)	−51.33 [−193.40, 90.73] *p =* 0.479
Professional Attendances	740.62 (600.80)	961.04 (1150.52)	−220.42 [−528.91, 88.07] *p =* 0.161
Therapeutic procedures	260.29 (687.27)	439.06 (1148.35)	−178.77 [−513.60, 156.06] *p =* 0.295
BoNT-A procedures	47.36 (120.20)	41.19 (118.25)	6.17 [−34.19, 46.52] *p =* 0.765
Pharmaceuticals	2736.29 (4414.67)	3018.04 (4377.43)	−281.75 [−1751.98, 1188.47] *p =* 0.707

Notes: Compete cases = cases with complete data over 12 months; OT and PT = occupational therapy and physiotherapy.

**Table 6:** Primary and secondary analysis of Incremental Cost-Effectiveness Ratios over 12 months (all costs in Australian Dollars).

Variable	Intervention	Control	Difference [Bootstrapped 95% CI], *p*-Value
*Base Case* (*Imputed Values*, *n* = *140*)			
Costs over 12 months	$12,233.26	$7122.22	$5111.04 [$1892.19, $8329.90] *p* = 0.02
QALY gain over 12 months	0.58	0.54	0.04 [−0.03, 0.12] *p* = 0.266
ICER (AU $/QALY gain)	$63,974.11		
GAS over 12 months	41.22	41.00	0.22 [−4.69, 5.12] *p* = 0.930
ICER (AU $/GAS)	$1667.51		
*Sensitivity Analysis* (*complete cases at 12 months*, *n* = *53*)			
Costs over 12 months	$13,722.16	$5,849.12	$7,873.04 [$1,011.38, $14,734.7] *p* = 0.025
QALY gain over 12 months	0.64	0.56	0.08 [−0.01, 0.18] *p* = 0.095
ICER (AU $/QALY gain)	$65,792.80		
GAS over 12 months	42.21	39.45	2.75 [−4.69, 10.19] *p* = 0.468
ICER (AU $/GAS)	$1563.76		

Note: ICER: Incremental cost-effectiveness ratio (i.e., difference in costs divided by the difference in QALYs gained over the 12 month follow up period between the intervention and control groups).

## Data Availability

The data presented in this study are available on request from the corresponding author, as approval for raw data release has not been approved by the Ethics Committee.

## Supplementary Materials

The following supporting information can be downloaded at: https://www.mdpi.com/article/10.3390/toxins17070341/s1, Figure S1: Cost-effectiveness plane and CEAC for QALYs with complete case data, Figure S2: Cost-effectiveness plane for GAS with the complete case data; Table S1: Complete cases available by variable, timeframe, and group allocation.

## Abbreviations

The following abbreviations are used in this manuscript:

BoNT-A	Botulinum neurotoxin A
CI	Confidence interval
DOAJ	Directory of Open Access Journals
EQ-5D	Euroqol five dimension
EQ-5D-3L	Euroqol five dimension three level
GAS	Goal Attainment Scale
GP	General practitioner
HRQOL	Health-related quality of life
ICER	Incremental cost-effectiveness ratio
LD	Linear dichroism
MAR	Missing at random
MBS	Medicare Benefits Schedule
MICE	Multiple imputation by chained equations
MDPI	Multidisciplinary Digital Publishing Institute
OT	Occupational therapist
PBS	Pharmaceutical Benefits Scheme
PMM	Predictive mean matching
PT	Physiotherapist
QALYs	Quality-adjusted life years
SD	Standard deviation
SUR	Seemingly unrelated regression
UK	United Kingdom
US	United States of America
WTP	Willingness to pay
