# Supplementary material for: Economic Evaluation of the InTENSE Program of Therapy Alongside Botulinum Neurotoxin a for the Rehabilitation of Chronic Upper Limb Spasticity
Source: Toxins (Basel). 2025 Jul 4;17(7):341. doi: 10.3390/toxins17070341 (PMC12298870; doi:10.3390/toxins17070341)
Supplement: Supplementary file 1 [file toxins-17-00341-s001.zip › toxins-3659749-supplementary.pdf]

Supplementary Materials: Economic Evaluation of the InTENSE Program of Therapy Alongside Botulinum Neurotoxin A for the Rehabilitation of Chronic Upper Limb Spasticity

Rachel Milte, Jia Song, Sean Docking, Julie Ratcliffe, Ian D. Cameron, Maria Crotty, Louise Ada, Coralie English and Natasha A. Lannin

Table S1: Complete cases available by variable, timeframe, and group allocation.

Variable	*Source of Data*	*Time Frame*	*Participants with complete data n (%)*	
			Intervention	*Control*
Participant Demographics and Clinical Characteristics	Trial Records	Baseline	71 (100%)	69 (100%)
Intervention Therapist Time, Travel and Consumables	Intervention Records	Trial Period	71 (100%)	-
EQ-5D-5L	Trial Records	Baseline	71 (100%)	69 (100%)
		3 Months Follow Up	71 (100%)	67 (97%)
		12 Months Follow Up	68 (96%)	64 (93%)
Doctor visits, Laboratory tests, Radiology, Allied Health	Administrative Linked Data	Trial and Follow Up	66 (93%)	68 (98%)
Medications	Administrative Linked Data	Trial and Follow Up	66 (93%)	67 (93%)
Inpatient admissions, Emergency department presentations, Privately funded allied health outpatients, Aged care services.	Participant monthly diary	4 months	48 (68%)	51 (73%)
		5 months	45 (63%)	48 (70%)
		6 months	41(58%)	42 (61%)
		7 months	35 (49%)	39 (57%)
		8 months	30 (43%)	36 (52%)
		9 months	27 (38%)	33 (48%)
		10 months	27 (38%)	30 (48%)
		11 months	27 (38%)	30 (48%)

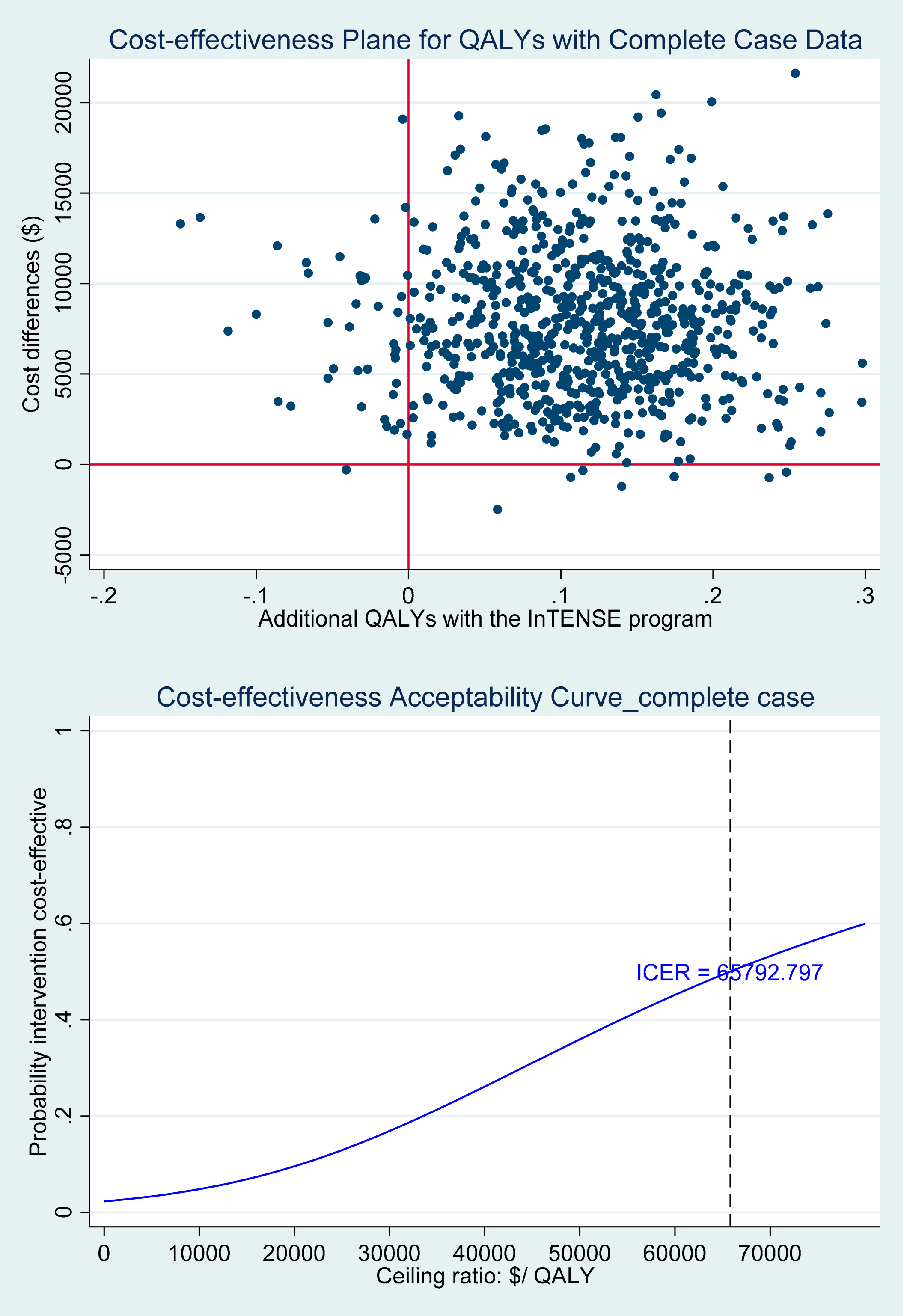

Figure S1: Cost effectiveness plane and CEAC for QALY with complete case data.

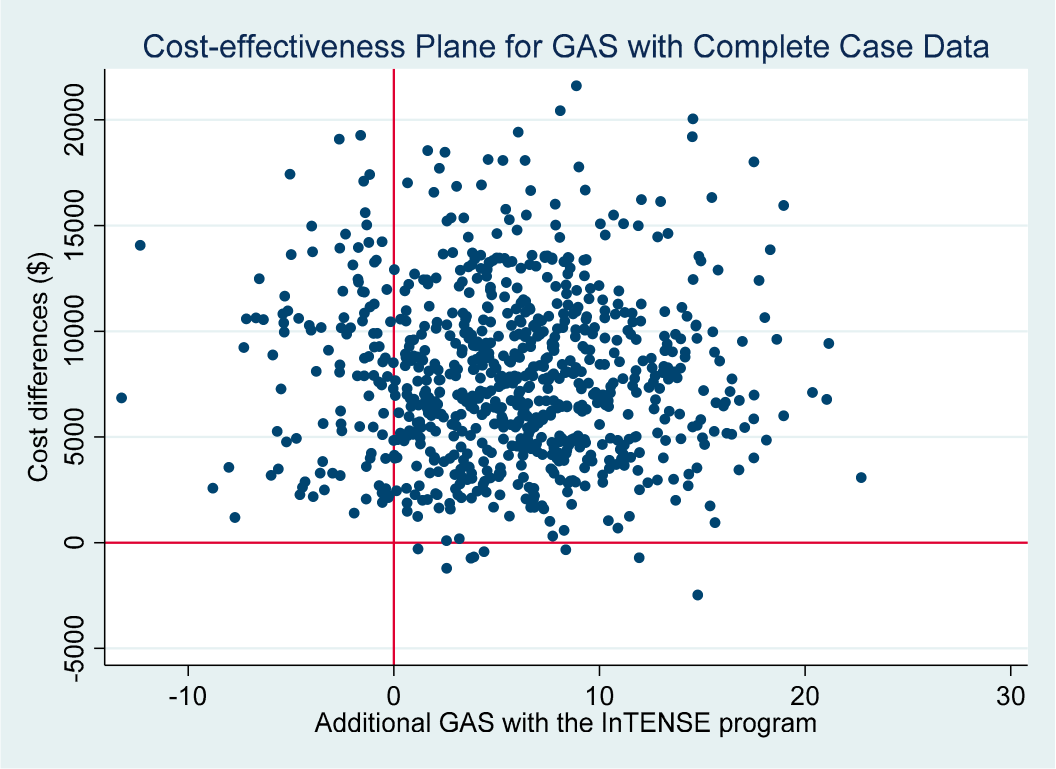

Figure S2: Cost effectiveness plane for GAS scores with complete case data.
